# Circulating extracellular vesicles across bovine gestation conserve characteristics independently of fetal age and sex

**DOI:** 10.3389/fvets.2026.1797667

**Published:** 2026-06-18

**Authors:** Maria Camila Lopez-Duarte, Daniella Heredia, Mauro Venturini, Jose Infante, Bruna L. C. Catussi, Angela Gonella-Diaza

**Affiliations:** 1 North Florida Research and Education Center, Institute of Food and Agricultural Sciences, University of Florida, Marianna, FL, United States; 2 Department of Animal Reproduction, Faculty of Veterinary Medicine and Animal Science, University of São Paulo, São Paulo, Brazil

**Keywords:** biomarker, cattle, extracellular vesicles, fetal sex, pregnancy

## Abstract

**Introduction:**

Extracellular vesicles (EVs) are released by cells into extracellular space and play a key role in intercellular communication. During pregnancy, embryo- and placenta-derived EVs are present in the maternal circulation, where they can serve as biomarkers of pregnancy status.

**Methods:**

The present study aimed to characterize the circulating EVs in pregnant cows carrying male or female calves. Crossbred Angus cows were subjected to Timed- Artificial Insemination (TAI) using the 7-Day CoSynch + CIDR protocol. Thirty days after TAI, pregnancy diagnoses were performed using gray mode ultrasonography, and thirty pregnant cows were randomly selected for the experiment. Blood samples were taken at days 45, 60, 90, 120, 150, 180, 210, 240, and 270 of gestation. Samples from each cow were centrifugated to separate the plasma, and 1 mL aliquots were stored at −80 °C. After parturition, calf sex was recorded, and samples from five male-carrying gestations (MCG) and five female-carrying gestations (FCG) were selected for EVs analysis.

**Results:**

EVs mean size and concentration were measured using nanoparticle flow cytometry and electron microscopy. Capillary wester analysis was used to corroborate the presence of EVs surface proteins CD63, CD9, CD81, and TSG101, confirming the presence of EVs in the isolates. The observed size ranged from 50 to 200 nm, with an average size of 87–105 nm, classifying the majority as small extracellular vesicles (sEVs). EVs size was not affected by fetal sex or gestation day. EVs concentration did not follow a clear pattern throughout pregnancy, and no significant effect of fetal sex or gestational day was observed.

**Conclusion:**

Most circulating EVs found in the plasma of pregnant of beef cattle, regardless of the fetal sex, are primarily sEVs. No differences were observed between male- and female-carrying gestations in EVs size or concentration throughout gestation.

## 1 Introduction

Extracellular vesicles (EVs) are nanoparticles with a bilipid layer. They are released by cells into extracellular space, and have an essential role in intercellular communication, serving as transport for proteins, lipids, RNA, microRNA (miRNA), and DNA (1). The International Society for EVs (ISEV) classifies these nanoparticles based on size, small EVs (sEV; < 200 nm) and large EVs (lEVl; >200 nm), as well as by their biogenesis, including exosomes, ectosomes, microvesicles, and apoptotic bodies. Regardless of their origin, all EVs carry surface receptors that enable communication with nearby or distant cells, influencing their phenotype or regulating cellular functions (2). Two-way EVs trafficking between the embryo and the mother occurs very early in pregnancy. These particles participate in conceptus implantation and can regulate blastocyst development as well as mediate the local endometrial immune system (3, 4).

Throughout recent years, it has been demonstrated that EVs can carry information to cells different from their parental line and even have the capacity for paracrine and intra-organ communication in humans (5) and mice (6). Subsequently, it has been demonstrated that EVs derived from embryonic tissue are present in the mother’s bloodstream in humans (4), mice (7), sheep (8), and cattle (9) causing a rise in the research of circulating EVs as biomarkers during pregnancy.

In bovines, it has been established that EVs play an essential role in the crosstalk between the embryo and the endometrium (9, 10). This crosstalk is crucial for maternal recognition of pregnancy and implantation (3, 11). Recently, it has been elucidated that EVs rapidly travel from the uterine environment into the bloodstream and have a short half-life in circulation (12). Although research on EVs during cattle gestation has increased in recent years, these studies have primarily focused on early pregnancy, leaving a significant gap in our understanding of EVs behavior beyond the first trimester. Zhao et al. (13) measured total circulating exosome concentrations and used a placental alkaline phosphatase (PLAP) antibody to identify those of placental origin at 60, 150, and 240 days of gestation. They reported that both total exosome and circulating placental exosome concentration significantly increased when comparing 60 and 150 days and 150 and 240 days of gestation. However, in this study, it is unclear whether the same animals were followed longitudinally across all time points. Nevertheless, these findings align with human data reported by Barnes et al. (14), who conducted a systematic review of circulating EVs during pregnancy. Their analysis showed that EVs concentrations generally increase as gestation progresses and placental mass expands, although they also highlighted substantial heterogeneity among studies, particularly regarding sample processing and EVs isolation methodologies. In contrast, other studies have not demonstrated a consistent increase in circulating EVs concentration during gestation. For example, Orozco et al. (15) reported that the number of total micro-particles (MPs) /mL increased from 9.3 × 10^6^ MPs/mL during the first trimester to 18.3 × 10^6^ MPs/mL during the second trimester and finally to 23.0 × 10^6^ MPs/mL during the third trimester of human pregnancy, however these numerical differences were not statistically significant. Buca et al. (16) reported no differences in the median concentration of platelet- or endothelial-derived EVs across the first, second, and third trimesters in pregnant women. And Lok et al. (17) observed a decrease in circulating MPs at 12 weeks of gestation followed by a return to normal levels in all the subsequent gestational timepoints (gestation weeks: 20, 24, 28, 32, and 36) that were not statistically different than samples collected 6 weeks after parturition in women.

These inconsistencies in circulating EVs concentration during gestation are also evident in animal models. Sheller-Miller et al. (18) indicated that average exosome concentration increased significantly throughout gestation (embryonic day 5 through 19) compared to the NP state with the highest exosome concertation reach at day 18. Still, exosome concentration per pup was significantly higher on embryonic day 18 compared to 9 and 13 when adjusted for the number of pups. In contrast, Zierden et al. (19) observed higher EVs concentrations in pregnant versus NP mice, however the circulating EVs concentration was higher at embryonic day 12.5 and 18.5 than during day 15.5, suggesting that this increment was not constant during time. Importantly, in both studies, animals were euthanized at each time point, meaning that comparisons were made across different individuals rather than longitudinally within the same animals. Collectively, these findings suggest that data regarding progressive changes in circulating EVs concentrations during gestation remain inconclusive, particularly when based on widely spaced time points or cross-sectional designs. Therefore, a more detailed characterization of circulating EVs across the full gestational timeline in cattle would be valuable to strengthen this observation and support broader conclusions.

In addition to the need for further and more detailed research on circulating EVs characteristics throughout pregnancy, a growing interest in the applicability of biomarkers-like EVs in the cattle industry has arisen in recent years. Finding a reliable marker to establish the fetal sex of a pregnant cow is a growing need in the market (20). Today, the only commercially available method for sex determination is transrectal ultrasound between 60 and 85 days of gestation (21). Nonetheless, the technique is not commonly adopted due to the lack of veterinarians who offer the service and the small window in which a diagnosis can be made. Alternatives such as noninvasive fetal sex determination using Cell-Free Fetal DNA (cffDNA) to amplify Y-chromosome-specific genes have been proposed by several research groups (reviewed in 22). However, the low concentration of cffDNA in the circulation of pregnant cows limits the feasibility of this approach (23).

Sexual dimorphism has been observed in several aspects of early embryonic development in humans (24) and other mammals such as mice (25), sheep (26), and cattle (27). The principal differences in cattle include variations in development rate (28), cell death (29), metabolism (30), and DNA methylation (31). Further along in pregnancy, other major differences between male and female carrying gestations in cattle have been determined. Pregnancies carrying male fetuses last longer and male offspring consistently have increased birth weights, heart girth, abdominal girth, and placenta efficiencies (32–34). Given that EVs participate in communication mechanisms and are released into circulation by the mother, embryo, and placenta during gestation, the longitudinal characterization of these particles may serve as a starting point for future investigations into embryo–maternal communication. Therefore, we hypothesize that circulating EVs size and concentration differ throughout pregnancy between male and female carrying gestations. Our objective is to gain a more comprehensive understanding of EVs dynamics during pregnancy and their potential use to differentiate between male and female carrying gestations.

## 2 Materials and methods

### 2.1 Ethics

This experiment was conducted at the North Florida Research and Education Center (NFREC), University of Florida, Marianna, FL. All animal procedures were approved by the Institutional Animal Care and Use Committee of the University of Florida (IACUC202300000159).

### 2.2 Animal handling, estrus synchronization, and timed-artificial insemination

During the breeding season of 2023, a total of 95 multiparous cycling, non-pregnant, and lactating Angus crossbred cows [*Bos taurus × Bos indicus;* age = 9.7 ± 0.4 yr.; Body Weight (BW) = 560 ± 8.3 kg] were enrolled in the study. All cows were kept in outdoor pens under grazing conditions with *Bahia grass* and fed *Bermudagrass* hay to meet nutritional requirements. Additionally, they had ad libitum access to fresh water and mineralized salt during the study. After clinical examination, cows with no signs of disease were enrolled in a 7-day CoSynch + CIDR split-time AI (TAI) protocol (35) using conventional semen from a single bull. At day −10 relative to TAI, and intramuscular injection of 100 μg gonadorelin (GnRH analog; Cystorelin, Boehringer Ingelheim, Duluth, GA, United States) and an intravaginal progesterone device (CIDR; Eazy-Breed CIDR, Zoetis, Lincoln, NE, United States) was inserted. At day −3, CIDR was removed and 25 mg of dinoprost tromethamine (Analog of prostaglandin F_2α_ [PGF_2α_]; Lutalyse HighCon, Zoetis, Lincoln, NE, United States) intramuscular injection was administered. Additionally, an estrous detection patch (Estrotect™ Heat Detection Aid. Rockway Inc., Spring Valley, WI, United States) was placed between the tail head and the hip in all the cows, as per manufacturer instructions. After 48 h of the CIDR removal, estrous detection patch scores were recorded. The cows that presented more than 50% of the patch rubbed off were considered in estrous and inseminated 12 h later. TAI was performed 66 h after CIDR removal in cows that did not present signs of estrus. Pregnancy diagnoses were conducted using transrectal gray mode ultrasonography (Esaote ultrasound, MyLab Delta Vet, 10–5 MHz transducer, at 60% gain) 30 days after timed artificial insemination (TAI).

**Figure 1:**
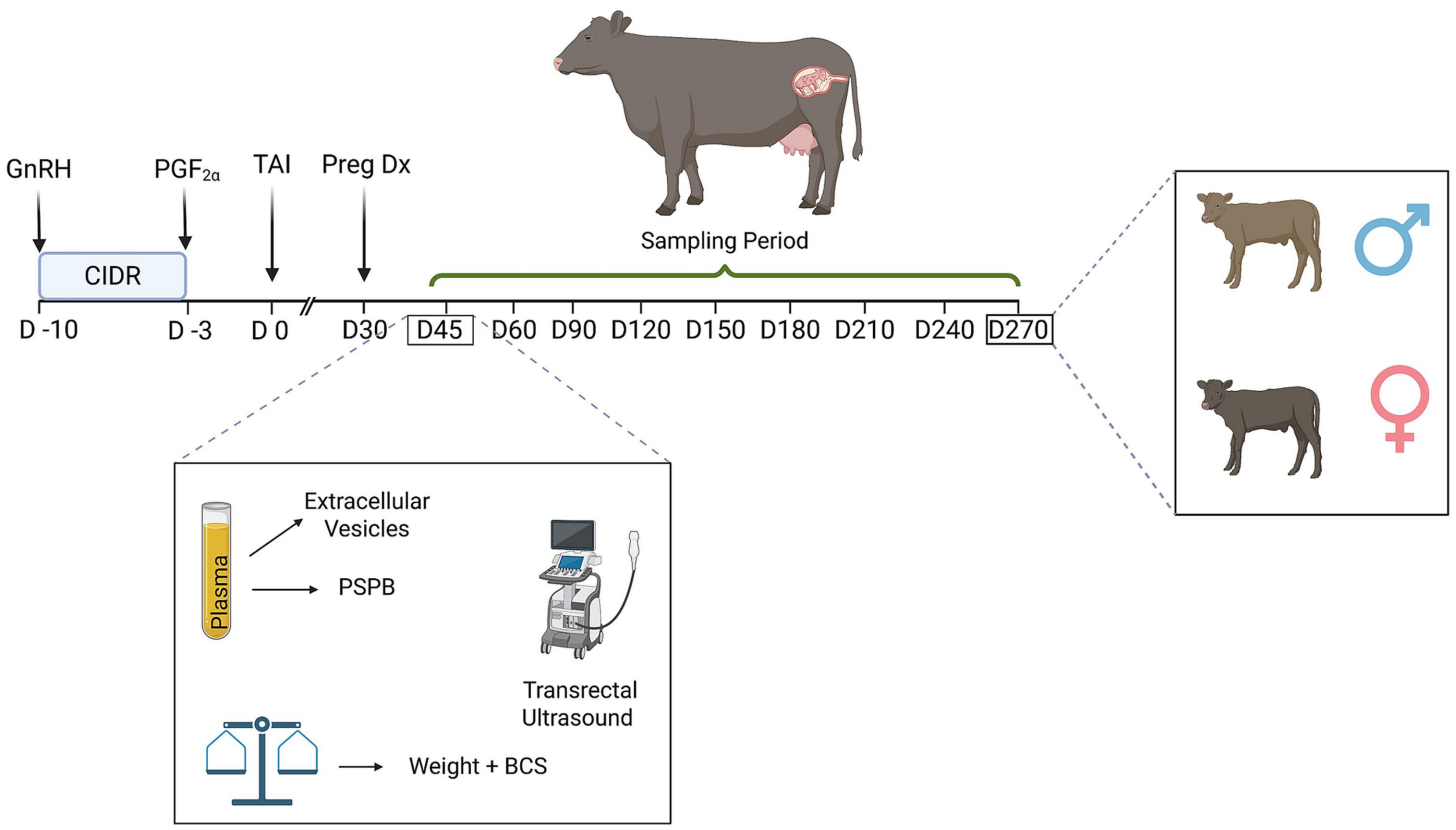
Experiment outline. Cows were subjected to a 7-day CoSynch estrous protocol, followed by timed artificial insemination (TAI). Thirty days after a transrectal ultrasound was performed for pregnancy diagnosis (Preg Dx). Two blood samples were collected once a month from day 45 to 270 for the isolation of extracellular vesicles (EVs) and the measurement of pregnancy-specific protein B (PSPB). During each collection day, body weight and body condition score (BCS) were documented. After calving, the sex of the offspring was identified and recorded. Created in BioRender. Lopez Duarte, M. C. (2026) BioRender.com/ieuwdgp. License:PL29OCEAGM Source: BioRender.

Thirty-nine of the 90 cows were diagnosed as pregnant, and 30 were randomly selected for the study. On days 45, 60, 90, 120, 150, 180, 210, 240, and 270 post-TAI (Figure 1), two blood samples were collected from the jugular vein using EDTA tubes (No. Cat. 367,863, BD Vacutainer, Becton, Dickinson and Co, NY, United States). Constantly thought the experiment, the first blood sample was always used for pregnancy-specific protein B (PSPB) determination and the second tube for EVs isolation. Finally, each day, transrectal ultrasonography evaluation of the uterine content was conducted to verify the viability of the pregnancy. Additionally, the BW and body condition score (BCS) were recorded each day. During the study, two cows had an abortion and were excluded from the data analysis. As part of routine management at NFREC, all cows were monitored through calving, calves were weighed, ear-tagged and have their sex recorded within 24 h of birth.

### 2.3 Blood samples processing

Immediately after collection, samples were placed on ice-cold water and transported to the laboratory (approximately, 15 min after last sample was collected). After arriving at the laboratory, blood samples for PSPB evaluation were centrifuged for 10 min at 3,000 RCF (Allegra X-22R Centrifuge, Beckman Coulter) to separate the plasma. Later, plasma was partitioned into aliquots of 2 mL and were stored at −80 °C. Blood tubes for EVs isolation were also centrifuged for 10 min at 3000 RCF, then plasma was partitioned into four different aliquots of 1 mL. To remove additional cellular nucleic acids, cellular debris, and avoid platelet contamination, a second centrifugation of the resulting plasma was performed for 10 min at 16,000 RCF (Microcentrifuge 24, USA Scientific) and supernatant was transferred to a new tube and stored at −80 °C until EVs isolation. After calving, the samples were retrospectively sorted by the sex of the calf. Next, samples from 10 cows, five male-carrying gestations (MCG) and five female-carrying gestations (FCG), were randomly selected to characterize the EVs throughout gestation and measure PSPB, this number of samples was the same in all time points, except on day 270 given that two cows (FCG) calved before the collection day.

### 2.4 Pregnancy-specific protein B concentration

Plasma Pregnancy-Specific Protein B concentration was measured in duplicate using ELISA (BioPRYN Flex; BioTracking LLC, Moscow, ID). As directed by the assay protocol, a standard curve ranging from 0 to 16 ng/mL was generated, and results were read at 640 nm with an AccuSkan plate reader (Thermo Fisher Scientific, Waltham, MA, United States). The intra-assay and inter-assay coefficients of variation were 1.65 and 12.1%, respectively.

### 2.5 Extracellular vesicles

The present study follows the nomenclature proposed by the MISEV2023 guidelines (36). Accordingly, the term EVs is used throughout this manuscript to refer to all lipid bilayer–enclosed particles released by cells. As defined by MISEV, EVs can be further classified as exosomes, micro-vesicles, or apoptotic bodies based on their biogenesis. However, several of the studies cited in this manuscript refer to these particles as “exosomes” or “microparticles” without providing direct evidence of their subcellular origin, as they were published prior to the release of the MISEV guidelines. For clarity and consistency, these particles are referred to as EVs throughout the present manuscript, except when a specific study is being cited.

#### 2.5.1 Extracellular vesicle isolation

Frozen plasma aliquots were thawed and sequentially centrifuged at 300 RCF, 2000 RCF and 10,000 RCF (Microcentrifuge 24; USA Scientific) for 10, 30, and 30 min, respectively. The supernatant was retained after each centrifugation step. The resultant supernatant was passed through a 0.22 μm filter (No. Cat. SLGSV255FEMD Millipore, Billerica, MA, United States) using vacuum filtration and transferred to pre-sterilized polycarbonate tubes (No. Cat. 355,622, Beckman Coulter, Brea, CA). Using a type 45 Ti rotor (Beckman Coulter Optima™ XE Ultracentrifuge), the samples were ultracentrifuged at 150,000 RCF for an hour at 4 °C. The supernatant was discarded, and the interior of the tube was rinsed with chilled sterile phosphate-buffered saline (PBS, pH 7.4). The pellet was then resuspended in 0.1–0.2 mL of PBS and transferred to 500 μL microtubes (No. Cat. EP0030124537, Eppendorf, Edison, NY, United States). The isolates were kept in dry ice during transportation to the Interdisciplinary Center for Biotechnology Research (ICBR) Cytometry Core. Size and concentration were measured immediately after arrival.

#### 2.5.2 Extracellular vesicles particle count and size

A NanoFCM Flow Nanoanalyzer (nFCM; NanoFCM Inc., Nottingham, United Kingdom) was used to measure particle count/concentration and size distribution. Before each use, the instrument was calibrated using one population of silica nanoparticles Cocktail (S16M-Exo: 40–200 nm, NanoFCM) as an internal control and to generate a sizing calibration curve. The particle density standard used in the cocktail was a population of nanoparticles provided at a concentration of 2.17 × 10^10^ particles mL^−1^ and diluted 1:100. The sizing standard contained four populations of nanoparticles ranging from 40 to 200 nm, chosen to cover the expected particle size range.

All samples were initially diluted with PBS in a 1:40 ratio; further dilutions were made to accommodate the device’s optimal detection range (∼1 × 108 particles mL − 1) if needed. The Fluorescein isothiocyanate (FITC) intensity threshold was automatically set by the instrument as a “small threshold” setting which was applied consistently across all data analyses. Once the appropriate dilution was determined, each sample was passed and recorded through the nFCM detector for 1 min with a flow pressure of 1.0 kPa, and data were analyzed by the NanoFCM Software (V1.17 NanoFCM Inc., Xiamen, China). EVs concentration data were first analyzed as reported in the NanoFCM output as particles per mL of plasma. In addition, a second analysis was performed by normalizing EVs concentration to each cow’s body weight at the time of sampling and the corresponding plasma volume. The normalization was calculated using the following equation (37):


$$\text{Normalized}\;\mathrm{EVs}=\frac{\mathrm{EVs}\;\text{Concentration}*\text{Plasma Volume}}{\text{Body Weight}}$$

Both analyses are presented in the results section of this article.

#### 2.5.3 Extracellular vesicles transmission electron microscopy

Glow-discharged 400 mesh carbon-coated Formvar copper grids (No. Cat. FCF400CU-UB, Electron Microscopy Sciences, Hatfield, PA) was floated onto 5 μL of suspension for 5 min. Then transferred to a drop of deionized water for 5 s, the excess solution was blotted from the grid with filter paper. The sample grid was floated onto a drop of 1% aqueous uranyl acetate for 30 s, blotted dry, and examined with a FEI Tecnai G2 Spirit Twin TEM (FEI Corp., Hillsboro, OR). The digital images were acquired with a Gatan UltraScan 2 k × 2 k camera and Digital Micrograph software (Gatan Inc., Pleasanton, CA).

#### 2.5.4 Extracellular vesicle capillary simple western analysis

A pool of isolated EVs was prepared using 5 μL of one MCG and one FCG sample for each collection time point, along with 10 μL of PBS. The EVs in the pooled sample were lysed in 50 μL 1 × RIPA buffer, and the protein extract samples were centrifuged at 12,000 RCF for 30 min at 4 °C. This pooling approach was used to demonstrate the presence of canonical EVs markers in the isolates, in accordance with MISEV2023 guidelines (36), and was not intended to support comparative analyses between gestational time points or between MCG and FCG. The EVs marker analysis was performed using the Simple Western system from Protein Simple (Biotechne Ltd), using a 12–230 kDa separation module and 13-capillary cartridge. Each well contained 3 μL of sample, with concentrations ranging from 1.2 to 0.6 μg. The primary antibodies used to detect target proteins were anti-CD63 (1:10 dilution), anti-CD81 (1:50 dilution), anti-CD9 (1:10 dilution), anti-TSG101 (1:50 dilution), and anti-Calnexin (1:100 dilution). CD63, CD81, and CD9 are well-established tetraspanins commonly used as canonical EVs markers (38, 39), while TSG101 is an endosomal protein frequently used to confirm the presence of exosome-associated vesicles (40, 41). Additionally, calnexin is a standard negative marker for EVs purity, used to confirm the absence of endoplasmic reticulum contamination in the isolate (42). The 12–230 kDa plate was run with an electrophoresis Jess instrument (ProteinSimple, San Jose, CA) and programmed with a separation load time of 200 s, stacking load time 15 s, sample load time 9 s, separation time 25 min, separation voltage 375 V, blocking time 30 min, primary antibody time 30 min and second antibody time 30 min. Protein signal intensity, peak area, and molecular weight were analyzed with Compass SW Software (Bio-Techne, United States).

### 2.6 Data analysis

All data were analyzed as a completely randomized design with repeated measures using GLIMMIX in SAS (version 9.4; SAS Institute Inc., Cary, NC, United States). The model included fetal sex, day of gestation, and their interaction (fetal sex × day) and cow was included as a random effect, with fetal sex nested within cow to account for individual variability. The BW and BCS were accounted as covariates. Nonetheless, the use of either of these covariates had no significant effect on the model and, as a result, were not accounted for in the final model. Studentized residuals (SR) plots and the Shapiro–Wilk test were used to assess normality and homoscedasticity of the residuals. The size and concentration of EVs were log-transformed to normalize the data, and results are described as back-transformed. Unless otherwise stated, the results of all variables are presented as arithmetic means ± standard error of the mean (SEM). Statistical significance was set as *p* ≤ 0.05, and trends between 0.05 < *p* ≤ 0.10. Results were plotted using GraphPad Prism (version 10.4.1; GraphPad Software, Boston, MA, United States).

## 3 Results

Of the 95 multiparous Angus cows that were synchronized and inseminated, 39 (41.05%) were diagnosed as pregnant, from which 30 were randomly selected for the study. During the study, two cows underwent spontaneous abortion or experienced fetal death between days 60 and 150 of gestation and were excluded from the statistical analysis. Thirteen of the total calves were male and 15 were female (MCG: n = 13; FCG: *n* = 15).

**Figure 2:**
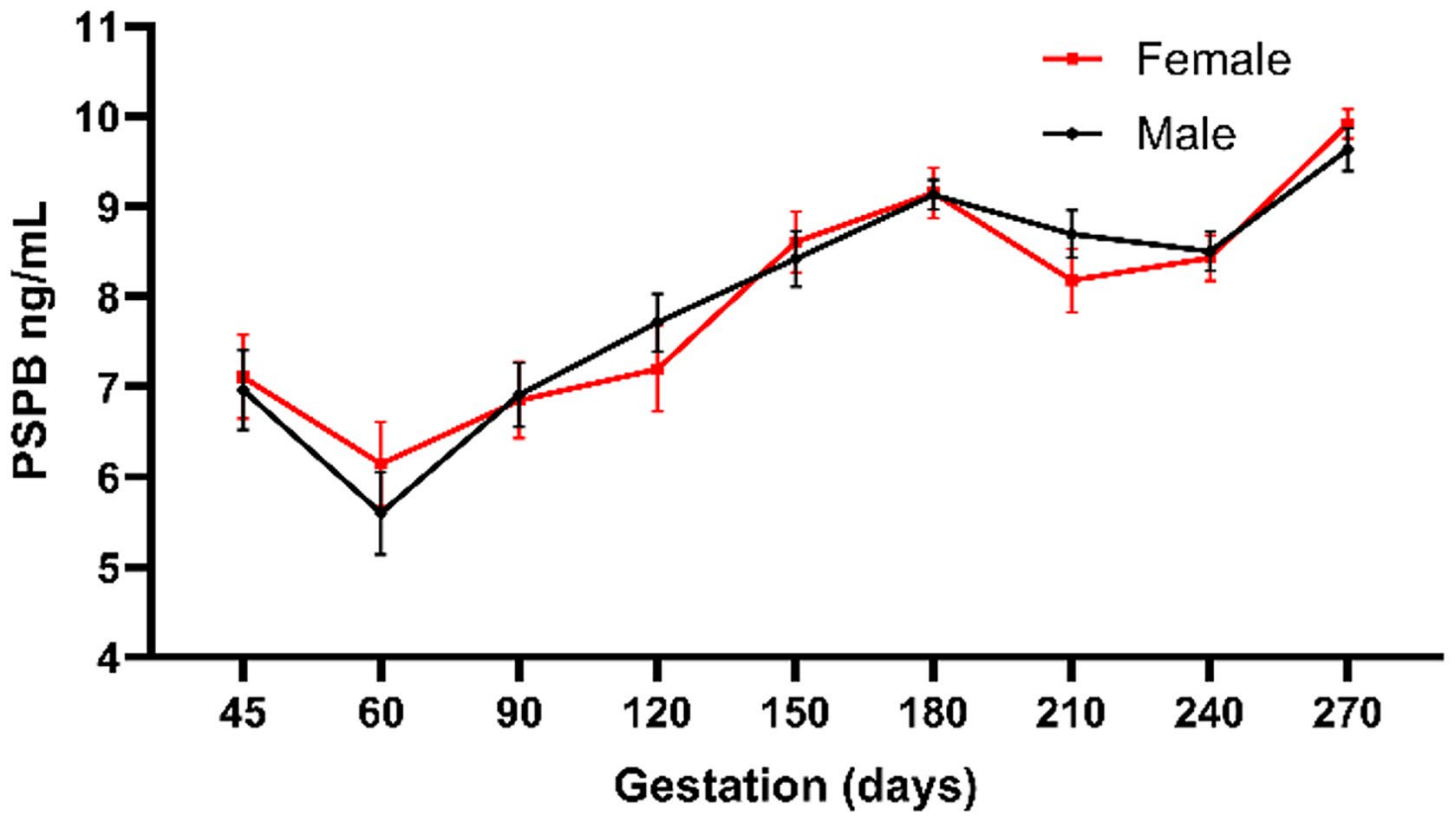
Circulating concentration of pregnancy-specific protein B (PSPB) ng/mL in male carrying gestation (MCG, *n* = 5) and female carrying gestation (FCG, *n* = 5) cows throughout the whole gestation. Day 270 had 5 MCG and 3 FCG since two cows calved earlier. Error bars show SEM. Created with GraphPad Prism 11.

The plasma PSPB concentration demonstrated the expected temporal trend increasing throughout pregnancy, with a clear peak before calving. The plasma PSPB concentration from MCG and FCG is shown in Figure 2. There was no statistically significant difference (*p* = 0.96) in the concentration of PSPB between MCG and FCG throughout gestation and no interaction (*p* = 0.74) in PSPB concentration between fetal sex and day of gestation was observed.

**Figure 3:**
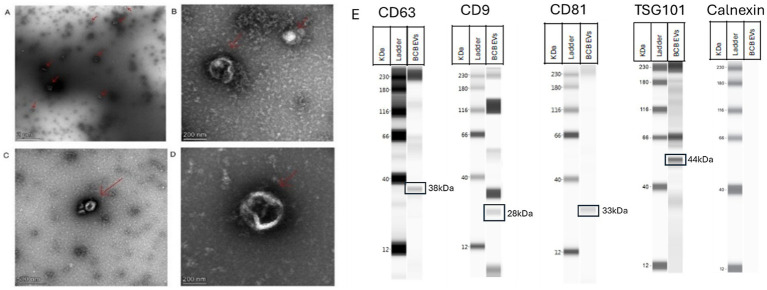
Characterization of extracellular vesicles (EVs) of pooled samples of isolated EVs from both male carrying gestation (MCG, *n* = 5) and female carrying gestation (FCG, *n* = 5) from day 45 to 270. **(A)** Representative TEM images of EVs. Scale bar, 2 μm. **(B)** Scale bar, 200 nm. **(C)** Scale bar, 500 nm. **(D)** Scale bar, 200 nm. **(E)** Representative bands of automated capillary simple western for EVs markers (CD9, CD63, CD81, TSG101, and negative control Calnexin).

EVs from MCG and FCG were characterized morphologically and molecularly according to the recommendations set by the ISEV (36). We used pooled aliquots from different sampling days and cows for TEM imaging (Figures 3A–D). The resulting images demonstrate the presence of EVs, which appear as spherical lipid bilayer particles with size variations ranging from 50 to 250 nm, confirming the presence of sEVs (Figures 3B,D).

The capillary simple western analysis showed that proteins CD63, CD9, CD81, and TSG101 were presented in EVs from pooled samples of MCG and FCG. Additionally, those EVs isolates had no presence of endoplasmic reticulum protein calreticulin indicating the presence of EVs in our samples and further confirming the successful isolation of EVs (Figure 3E).

**Figure 4:**
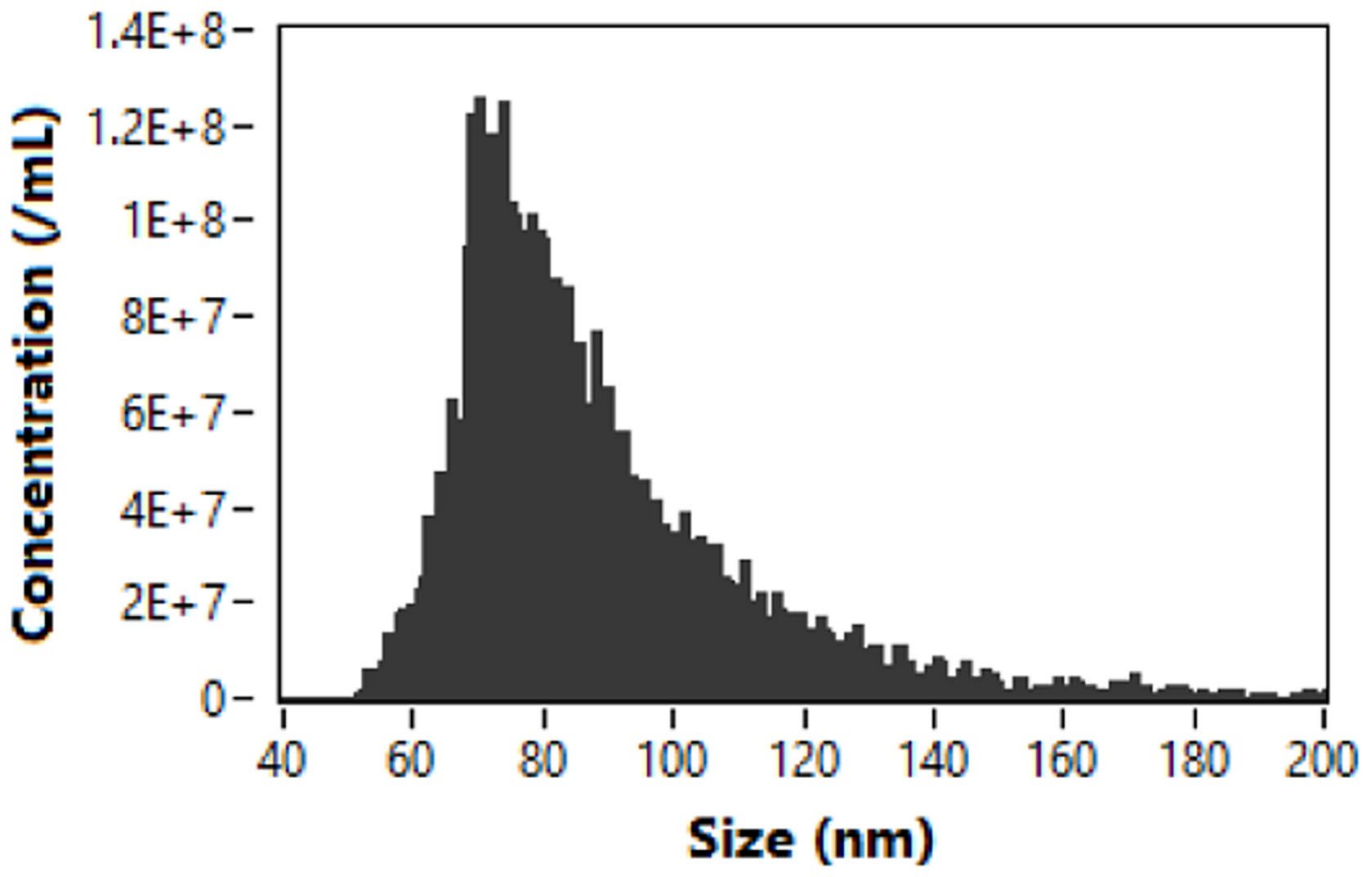
Representative profile of size-distribution of EVs isolated from plasma of both both male carrying gestation (MCG) and female carrying gestation (FCG) by NanoFCM.

**Figure 5:**
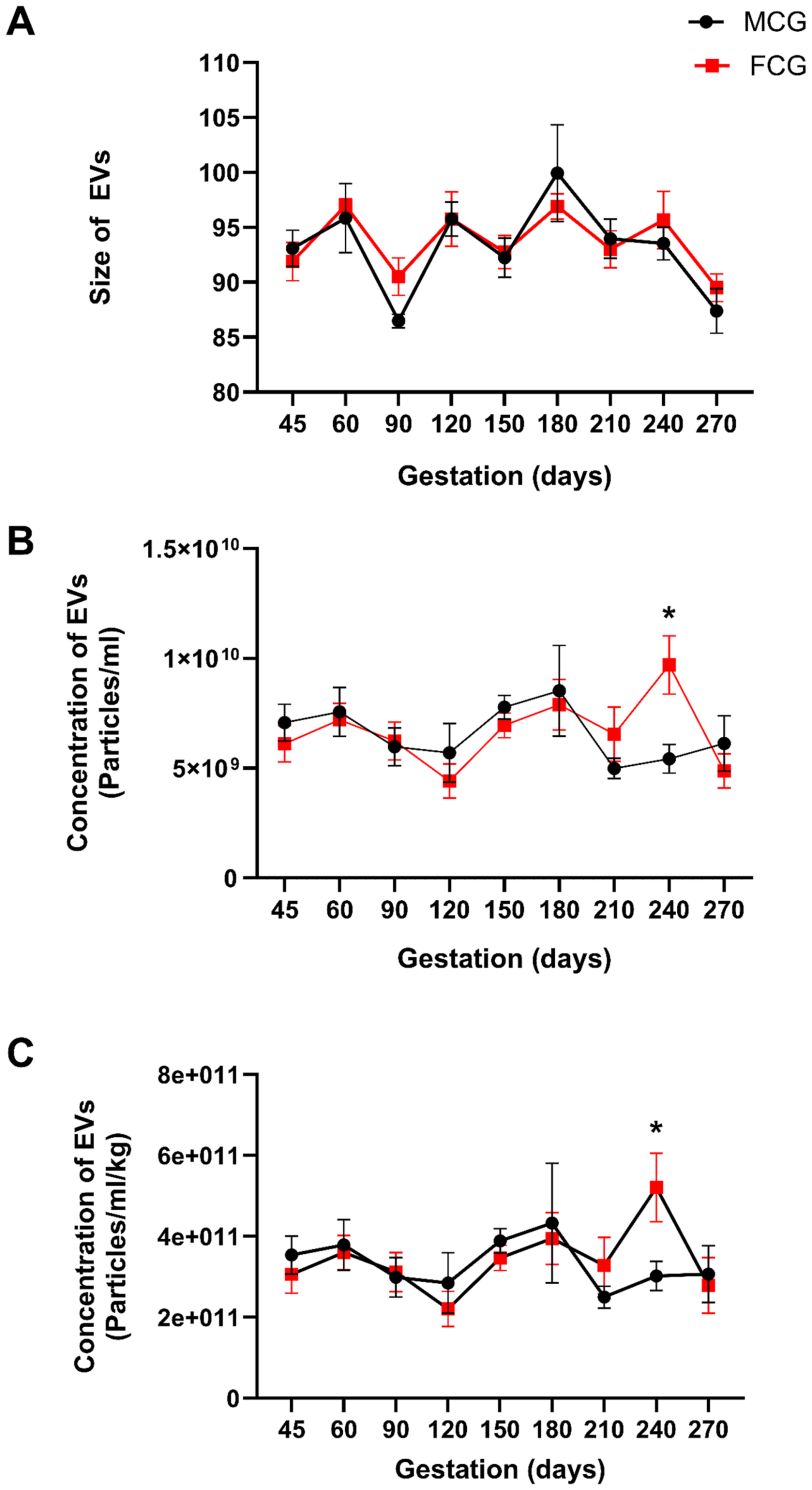
Size and concentration data measured through NanoFCM. **(A)** Circulating extracellular vesicle average size (nanometers) throughout the whole pregnancy in cows with a male carrying gestation (MCG, *n* = 5) and female carrying gestation (FCG, *n* = 5). Day 270 had 5 MCG and 3 FCG since two cows calved earlier. **(B)** Extracellular vesicle concentration (particles/mL); **(C)** (particles/mL/kg); in plasma samples during pregnancy in cows with MCG and FCG. Error bars show SEM, and an asterisk represents *p* ≤ 0.05. Created with GraphPad Prism 11.

To determine the size distribution and particle concentration, samples from five MCG and five FCG at each time point were analyzed by nFCM, with the exception of day 270, when only three FCG samples were available given two cows had already calved. The size analysis of the EVs revealed that the particles in all samples were distributed within a size range of 50–200 nm (Figure 4). The average particle size across samples ranged from 87 to 105 nm (Figure 5A) and the size distribution peaked at approximately 87.5 nm (Figure 4). Given these measurements, the EVs population falls into the category of sEVs, characterized by the size of less than 200 nm, and lEVs (Figure 3D) for more than 200 nm (36). The mean size of the EVs throughout gestation between MCG (92.89 ± 1 nm) and FCG (93.83 ± 0.7 nm) showed no statistically significant difference (*p* > 0.05). Similarly, day of gestation did not affect the size of EVs (Figure 5A).

As described in the methodology section, EVs concentrations at each time point were analyzed using two approaches. First, the EVs concentration was analyzed as particles per mL of plasma and later this value was normalized by body weight and estimating the plasma volume at each sampling day. The mean concentration for all samples throughout gestation was 6.70 × 10^9^ ± 2.71 × 10^9^ particles/mL. As shown in Figure 5B, EVs concentrations in both MCG and FCG groups showed no clear increasing or decreasing pattern throughout gestation. The concentration remains similar between both groups for all sampling dates and was only different on day 240 (*p* = 0.025), where MCG had a lower concentration of EVs (5.43 × 10^9^ ± 7.48 × 10^8^ particles/mL) in comparison with FCG (9.7 × 10^9^ ± 1.49 × 10^9^).

The mean concentration of EVs when normalized by body weight and plasma volume was 3.35 × 10^11^ ± 1.47 × 10^10^ particles/mL/kg (Figure 5C). Similarly to the concentration of EVs measured as particles/mL, no pattern of increasing or decreasing concentration throughout gestation was observed (Figure 5B). After normalization, MCG (3.02 × 10^11^ ± 3.6 × 10^10^, *p* = 0.025) also presents a lower concentration of EVs/mL/kg than FCG (5.21 × 1,011 ± 8.46 × 1,010, *p* = 0.025) at 240 days. Similarly, on all other days of gestation, there was no difference in EVs concentration between MCG and FCG.

## 4 Discussion

Identifying reliable biomarkers of pregnancy status and fetal characteristics remains an ongoing challenge in cattle reproductive management. A better understanding of the circulating biomarkers that change during gestation is important for filling existing knowledge gaps in reproductive biology and for contributing to the development of new biotechnologies that can improve reproductive efficiency in beef production systems. The use of transrectal ultrasonography for fetal sex determination can offer economic advantages when marketing pregnant females (20). However, this method requires specialized equipment, a trained veterinarian, and must be performed within a narrow time window. These factors limit their widespread use among producers. This study aimed to evaluate whether circulating biomarkers such as PSPB and EVs, differ between MCG and FCG pregnancies, potentially offering a more accessible alternative for fetal sex determination.

Concentrations of PSPB across gestation exhibited a progressive increase, rising from approximately 6 ng/mL at day 60–8.5 ng/mL by day 150, and reaching a pronounced peak of 9.5 ng/mL just before parturition. These trends are consistent with previous reports in both dairy and beef cattle (43–46). Griffin et al. (46) observed rising levels of early secreted PAGs (Ab 45, by in-house sandwich ELISA), reporting 20 ng/mL at day 60, increasing to 60 ng/mL by day 210, and peaking near parturition at 150 ng/mL. However, our observations showed no statistically significant differences in plasma PSPB concentrations between MCG and FCG at any day of gestation. Contrasting results have been reported by other authors. For instance, Lobago et al. (47) reported that PAGs (classical heterologous radioimmunoassay [RIA-497]) concentrations in serum between days 60 and 100 of gestation differed between FCG (16.9 ± 1.9 ng/mL) and MCG (10.1 ± 1.9 ng/mL). However, in their study, variation in PAGs levels also depended on breed, comparing Borana × Holstein-Friesian crossbreds with pure Borana cattle. Similarly, Zoli et al. (45) reported that PAGs (RIA497) concentrations varied according to both fetal sex and breed. In Holstein cows, MCG exhibited higher levels (3 ng/mL) than FCG (1 ng/mL) at day 260 of gestation, while in Hereford cows the trend was reversed, with FCG showing higher concentrations (4 ng/mL) compared to MCG (2 ng/mL). Interestingly, Doyle et al. (48) reported that FCG Holstein cows had lower PAGs (Commercial ELISA, IDEXX, Liebefeld-Bern, Switzerland) concentrations in milk between weeks 5 and 8, but this pattern shifted after week 14, when FCG began to show higher concentrations than MCG. Thus, variations in PAGs concentrations during pregnancy can be strongly influenced by factors such as breed, the specific PAGs, and the assay to measure them (49), as well as parity (50) making PAGs an unreliable marker for fetal sex determination.

The commercial application of cffDNA for fetal sex determination in cattle has been limited due to the low concentration of specific genes in maternal blood (23). Most of the cffDNA present in maternal circulation is encapsulated within EVs (51). Based on this, we explored whether characteristics such as EVs size and concentration could serve as markers for fetal sex differentiation. Present data showed no linear increase in EVs concentration throughout gestation, and no clear trend or consistent pattern was observed in EVs behavior except for a transitory difference between MCG and FCG at day 240. In contrast, Zhao et al. (13) reported an increase in circulating EVs concentration across gestation day 60 (2.5 × 10^10^ particles/mL), 150 (6 × 10^10^ particles/mL), and 240 (11 × 10^10^ particles/mL) of gestation. This pattern is consistent with some human data (Table 1), where EVs concentration rises though pregnancy (52, 53) going from about 5 × 10^11^ in early pregnancy to roughly 5 × 10^12^ by the third trimester (53). Similarly, a recent review (14) reported an increase in EVs concentrations when the first trimester was compared with the second, and when the first trimester was compared with the third. However, most studies found no difference between the second and third trimesters, with the exception of two studies that used alternative quantification methods (52, 53). In the other hand, no change on the concentration of EVs though pregnancy is also reported by several studies (Table 1) in humans (15–17). Similarly, observations in mice are not consistent across studies either. For instance, Sheller-Miller et al. (18) observed an increase in the EVs concentration when embryonic day 9 is compared to day 18 (*p* = 0.002) and day 13–18 (*p* = 0.004). In contrast, Zierden et al. (19) reported concentrations beginning at 2.3 × 10^11^ on embryonic day 12.5, a decrease to 1.8 × 10^11^ by day 15.5 and then increase slightly to 2.8 × 10^11^ by day 18.5, indicating no clear linear pattern of change in circulating EVs concentrations during gestation.

**Table 1:** Summary of previous studies that evaluated total circulating EVs and feto placental EVs, highlighting the variation in results across literature.

Species	1st–2nd trimester increment	Linear increment through pregnancy		References
		Total circulating EVs^1^	Feto-placental EVs	
Human	No	No	N/A	(16)
Human	No	No	No	(17)
Human	No	No	No	(15)
Human	Yes	No	No	(57)
Human	Yes	No	Yes	(53)
Human	Yes	Yes	Yes	(52)
Human	Yes	Yes	Yes	(58)
Human	Yes	N/A	N/A	(59)
Mice	–	No	N/A	(19)
Mice	–	Yes	N/A	(18)
Bovine	Yes	Yes	Yes	(13)
Bovine	No	No	N/A	Present study

^1^The present study was written in accordance with the MISEV (36) nomenclature guidelines that define EVs as lipid bilayer cell-derived particles and that are classified in Exosomes, micro-vesicles, or apoptotic bodies, according to their biogenesis. Still, several of the studies cited in this table refer to these particles as exosomes or microparticles without defining the biogenesis of their origin, as they were published prior to the release of the MISEV guidelines.

Although Zhao et al. (13) were the first to study the characterization of circulating EVs in cows at different days of gestation, they only considered three time points. In the present study, we analyzed EVs concentration at nine different time points of pregnancy, having a broader scale and a more comprehensive view of the fluctuations that occur from month to month. This broader analysis enabled us to determine the dynamic nature of the concentration and size of EVs throughout bovine gestation. In addition, the description of the methodology section of Zhao et al. (13) is insufficient to determine if samples collected from the different timepoints belong to the same cow. In the present study, the same group of cows was consistently followed throughout gestation, and therefore we can consistently compare EVs concentration from the same individual throughout gestation. These methodological differences may help account for the contrasting EVs concentration patterns between the two studies: the progressive gestational increase reported by Zhao et al. (13), compared with the relatively stable concentrations observed throughout gestation in the present study.

The present study is the first to take into consideration the fetal sex when analyzing the concentration and size of EVs in cattle. However, our data indicate that the size and concentration of EVs in cows are not associated with fetal age or sex throughout gestation. A transient difference was observed only at day 240, where MCG had fewer EVs concentration compared to FCG. this is consistent with the findings from Salomon et al. (52, 53) reported that fetal sex had no effect on the concentration of placental-derived exosomes in humans. Additionally, although no morphological characteristic was demonstrated to be affected by fetal sex, it is known that this can influence EVs cargo, such as cffDNA (54), miRNA (55), and proteins (56). While the cargo of EVs was not within the scope and, as a result, not evaluated in the present study, it will be important to consider this type of analysis in future studies that aimed to look for biomarkers of fetal sex in maternal circulation. Furthermore, the overall size of circulating EVs remained consistent throughout gestation, with no significant differences observed across fetal ages with an average particle size across samples of 87–105 nm. This finding is consistent with previous studies reporting similar EVs size distributions across species, regardless of pregnancy stage. In cows, average particle sizes ranged from 30 to 140 nm (13); in humans, from 85 to 115 nm (53) and in mice, from 80 to 140 nm (19).

It is important to address certain limitations of the present study. Notably, the EVs analyzed here represent the total population of circulating EVs, and no placental- or fetal-specific markers were used to identify EVs of feto-placental origin. Consequently, changes specifically attributable to feto-placental EVs may have been masked within the total circulating pool, which reflects contributions from multiple maternal and fetal sources whose relative proportions vary across gestation (Table 1). Additionally, as stated in the methodology and results EVs analyses were performed on a relatively small subset of animals (*n* = 5 MCG and *n* = 5 FCG), which limits the statistical power to detect subtle effects of fetal sex or gestational day, particularly at day 270 of gestation when two cows with FCG had already calved. Nevertheless, studies using larger cohort of samples and including feta/placental markers will be necessary to validate the presented data and to fully interpret the differences between circulating EVs in MCG and FCG. It is important to note, however, that EVs analyses were performed on samples collected longitudinally from the same cows throughout pregnancy, allowing each animal to serve as their own control and thereby reducing the influence of between-animal variability on the detection of gestational changes.

In conclusion, circulating EVs with similar morphological characteristics were depicted throughout the whole gestation, with similarities with other species like humans and mice. Additionally, no clear pattern was observed in the total concentration of circulating EVs through gestation and no effect of fetal sex was observed in any of the variables measured in this study, aside from a transient decrease in circulating EVs concentration in MCG at day 240. These findings collectively lead us to reject our initial hypothesis and suggesting that fetal sex may not have a significant influence on total circulating EVs concentration and morphological characteristics in cattle. Further research is needed to explore the underlying mechanisms governing EVs cargo and its differences between MCG and FCG during pregnancy. Moreover, further research must establish if EVs from placental origin show the same pattern as total circulating EVs shown in this study.

## Data Availability

The raw data supporting the conclusions of this article will be made available by the authors, without undue reservation.

## References

[1] György B Szabó TG Pásztói M Pál Z Misják P Aradi B . Membrane vesicles, current state-of-the-art: emerging role of extracellular vesicles. Cell Mol Life Sci. (2011) 68:2667–88. doi: 10.1007/S00018-011-0689-3, 21560073 PMC3142546

[2] Nazarenko I Rana S Baumann A McAlear J Hellwig A Trendelenburg M . Cell surface Tetraspanin Tspan8 contributes to molecular pathways of exosome-induced endothelial cell activation. Cancer Res. (2010) 70:1668–78. doi: 10.1158/0008-5472.CAN-09-2470, 20124479

[3] Kusama K Nakamura K Bai R Nagaoka K Sakurai T Imakawa K. Intrauterine exosomes are required for bovine conceptus implantation. Biochem Biophys Res Commun. (2018) 495:1370–5. doi: 10.1016/j.bbrc.2017.11.176, 29196267

[4] Giacomini E Alleva E Fornelli G Quartucci A Privitera L Vanni VS . Embryonic extracellular vesicles as informers to the immune cells at the maternal–fetal interface. Clin Exp Immunol. (2019) 198:15–23. doi: 10.1111/cei.13304, 31009068 PMC6718282

[5] Li Y Yin P Guo Z Lv H Deng Y Chen M . Bone-derived extracellular vesicles: novel players of Interorgan crosstalk. Front Endocrinol. (2019) 10:10. doi: 10.3389/fendo.2019.00846, 31920965 PMC6914759

[6] Tong M Stanley JL Chen Q James JL Stone PR Chamley LW. Placental Nano-vesicles target to specific organs and modulate vascular tone in vivo. Hum Reprod. (2017) 32:2188–98. doi: 10.1093/humrep/dex310, 29040541

[7] Sheller-Miller S Lei J Saade G Salomon C Burd I Menon R. Feto-maternal trafficking of exosomes in murine pregnancy models. Front Pharmacol. (2016) 7:7. doi: 10.3389/fphar.2016.00432, 27895585 PMC5108780

[8] Burns GW Brooks KE Spencer TE. Extracellular vesicles originate from the conceptus and uterus during early pregnancy in Sheep1. Biol Reprod. (2016) 94:56. doi: 10.1095/biolreprod.115.134973, 26819476

[9] Zhao G Yang C Yang J Liu P Jiang K Shaukat A . Placental exosome-mediated Bta-miR-499-Lin28B/let-7 axis regulates inflammatory bias during early pregnancy. Cell Death Dis. (2018) 9:704. doi: 10.1038/s41419-018-0713-8, 29899331 PMC5999645

[10] De Bem THC Bridi A Tinning H Sampaio RV Malo-Estepa I Wang D . Biosensor capability of the endometrium is mediated in part, by altered miRNA cargo from conceptus-derived extracellular vesicles. FASEB J. (2024) 38:e23639. doi: 10.1096/fj.202302423RR, 38742798

[11] Nakamura K Kusama K Bai R Sakurai T Isuzugawa K Godkin JD . Induction of IFNT-stimulated genes by conceptus-derived exosomes during the attachment period. PLoS One. (2016) 11:e0158278. doi: 10.1371/journal.pone.0158278, 27351483 PMC4924817

[12] Farias Fiorenza M Bridi A dos Santos G Maria Rosa P Alves L Germano Ferst J . Labeled extracellular vesicles can be found in the blood plasma shortly after intrauterine infusion in bovine. Anim Reprod. (2024) 21:e20240064. doi: 10.1590/1984-3143-AR2024-0064, 39286366 PMC11404864

[13] Zhao G Guo S Jiang K Zhang T Wu H Qiu C . MiRNA profiling of plasma-derived exosomes from dairy cows during gestation. Theriogenology. (2019) 130:89–98. doi: 10.1016/j.theriogenology.2019.03.001, 30878693

[14] Barnes MVC Pantazi P Holder B. Circulating extracellular vesicles in healthy and pathological pregnancies: a scoping review of methodology, rigour and results. J Extracell Vesicles. (2023) 12:e12377. doi: 10.1002/jev2.12377, 37974377 PMC10654380

[15] Orozco AF Jorgez CJ Ramos-Perez WD Popek EJ Yu X Kozinetz CA . Placental release of distinct DNA-associated Micro-particles into maternal circulation: reflective of gestation time and preeclampsia. Placenta. (2009) 30:891–7. doi: 10.1016/j.placenta.2009.06.012, 19692120 PMC2758063

[16] Buca D Lucidi A Buca DV Di Sebastiano F D’Angelo E Vespa S . Extracellular vesicles during the three trimesters of pregnancy. J Reprod Immunol. (2023) 159:103987. doi: 10.1016/j.jri.2023.103987, 37454539

[17] Lok CAR Van Der Post JAM Sargent IL Hau CM Sturk A Boer K . Changes in microparticle numbers and cellular origin during pregnancy and preeclampsia. Hypertens Pregnancy. (2008) 27:344–60. doi: 10.1080/10641950801955733, 19003636

[18] Sheller-Miller S Trivedi J Yellon SM Menon R. Exosomes cause preterm birth in mice: evidence for paracrine signaling in pregnancy. Sci Rep. (2019) 9:608. doi: 10.1038/s41598-018-37002-x, 30679631 PMC6345869

[19] Zierden HC Marx-Rattner R Rock KD Montgomery KR Anastasiadis P Folts L . Extracellular vesicles are dynamic regulators of maternal glucose homeostasis during pregnancy. Sci Rep. (2023) 13:4568. doi: 10.1038/s41598-023-31425-x, 36941297 PMC10027885

[20] Kim D Son M Jung D Heo S Kim M Yi J. Economic impacts of Ultrasonographic fetal sex determination on Hanwoo cattle profitability and market dynamics. Vet Sci. (2025) 12:201. doi: 10.3390/vetsci12030201, 40266948 PMC11946265

[21] Fricke PM Lamb C. Potential applications and pitfalls of reproductive ultrasonography in bovine practice. Vet Clin North Am Food Anim Pract.(2005). 419–36. doi: 10.1016/j.cvfa.2005.02.005 15955438

[22] Aucamp J van der Zwan H Geldenhuys Z Abera A Louw R van der Sluis R. Diagnostic applications and limitations for the use of cell-free fetal DNA (cffDNA) in animal husbandry and wildlife management. Res Vet Sci. (2023) 158:106–16. doi: 10.1016/j.rvsc.2023.03.013, 36989830

[23] Singh N Fernando C Hill JE Singh J Campbell J Dadarwal D. Identifying the minimum concentrations of cell-free fetal DNA in maternal blood required for bovine fetal sexing using PCR. Theriogenology. (2022) 191:192–9. doi: 10.1016/j.theriogenology.2022.08.015, 35998402

[24] Baines KJ West RC. Sex differences in innate and adaptive immunity impact fetal, placental, and maternal health. Biol Reprod. (2023) 109:256–70. doi: 10.1093/biolre/ioad072, 37418168

[25] Burgoyne PS. A Y-chromosomal effect on blastocyst cell number in mice. Development. (1993) 117:341–5. doi: 10.1242/dev.117.1.341, 8223257

[26] Catt SL O’Brien JK Maxwell WMC Evans G. Effects of rate of development of in vitro-produced ovine embryos on sex ratio and in vivo survival after embryo transfer. Theriogenology. (1997) 48:1369–78. doi: 10.1016/S0093-691X(97)00378-6

[27] Avery B Madison V Greve T. Sex and development in bovine in-vitro fertilized embryos. Theriogenology. (1991) 35:953–63. doi: 10.1016/0093-691X(91)90306-X, 16726963

[28] Abd El Naby WS Hagos TH Hossain MM Salilew-Wondim D Gad AY Rings F . Expression analysis of regulatory microRNAs in bovine cumulus oocyte complex and preimplantation embryos. Zygote. (2013) 21:31–51. doi: 10.1017/S0967199411000566, 22008281

[29] Ghys E Dallemagne M De Troy D Sauvegarde C Errachid A Donnay I. Female bovine blastocysts are more prone to apoptosis than male ones. Theriogenology. (2016) 85:591–600. doi: 10.1016/j.theriogenology.2015.09.050, 26506912

[30] Green MP Harvey AJ Spate LD Kimura K Thompson JG Roberts RM. The effects of 2,4-dinitrophenol and d-glucose concentration on the development, sex ratio, and interferon-tau (IFNT) production of bovine blastocysts. Mol Reprod Dev. (2016) 83:50–60. doi: 10.1002/mrd.22590, 26465354 PMC9006197

[31] Bermejo-Álvarez P Rizos D Rath D Lonergan P Gutierrez-Adan A. Epigenetic differences between male and female bovine blastocysts produced in vitro. Physiol Genomics. (2008) 32:264–72. doi: 10.1152/physiolgenomics.00234.2007, 17986520

[32] Ahmed RH Schmidtmann C Mugambe J Thaller G. Factors influencing calving difficulty and gestation length in dairy cows inseminated with beef sires. Animal. (2024) 18:101369. doi: 10.1016/j.animal.2024.101369, 39608181

[33] Dhakal K Maltecca C Cassady JP Baloche G Williams CM Washburn SP. Calf birth weight, gestation length, calving ease, and neonatal calf mortality in Holstein, Jersey, and crossbred cows in a pasture system. J Dairy Sci. (2013) 96:690–8. doi: 10.3168/jds.2012-5817, 23084888

[34] Redifer CA Duncan NB Meyer AM. Factors affecting placental size in beef cattle: maternal and fetal influences. Theriogenology. (2021) 174:149–59. doi: 10.1016/j.theriogenology.2021.08.015, 34454320

[35] Beef Reproduction Task Force (2025) Cow Protocols - 7-Day CoSynch + CIDR & split TAI. Available online at:https://beefrepro.org/cow-protocols/ (Accessed 20 July 2025).

[36] Welsh JA Goberdhan DCI O’Driscoll L Buzas EI Blenkiron C Bussolati B . Minimal information for studies of extracellular vesicles (MISEV2023): from basic to advanced approaches. J Extracell Vesicles. (2024) 13:e12404. doi: 10.1002/jev2.12404 38326288 PMC10850029

[37] Dalton RG Fisher EW. Plasma and blood volumes in Ayrshire cattle. Br Vet J. (1961) 117:115–9. doi: 10.1016/S0007-1935(17)43753-5

[38] Kowal J Arras G Colombo M Jouve M Morath JP Primdal-Bengtson B . Proteomic comparison defines novel markers to characterize heterogeneous populations of extracellular vesicle subtypes. Proc Natl Acad Sci USA. (2016) 113:E968–77. doi: 10.1073/pnas.1521230113, 26858453 PMC4776515

[39] Mamand DR Gustafsson O Sork H Wiklander RJ Bazaz S Liang X . Evaluation of tetraspanins in extracellular vesicle bioengineering. bioRxiv. (2026):699196. doi: 10.64898/2026.01.13.699196 PMC13098096 42022881

[40] Munir J Sadri M Zempleni J. Tsg101 knockout in the mammary gland leads to a decrease in small extracellular vesicles in milk from C57BL/6J dams and contributes to leakiness of the gut mucosa and reduced postnatal weight gain in suckling pups. J Nutr Biochem. (2025) 135:109782. doi: 10.1016/j.jnutbio.2024.109782, 39424203

[41] Colombo M Moita C van Niel G Kowal J Vigneron J Benaroch P . Analysis of ESCRT functions in exosome biogenesis, composition and secretion highlights the heterogeneity of extracellular vesicles. J Cell Sci. (2013) 126:5553–65. doi: 10.1242/jcs.128868, 24105262

[42] Hanson B Vorobieva I Zheng W Conceição M Lomonosova Y Mäger I . EV-mediated promotion of myogenic differentiation is dependent on dose, collection medium, and isolation method. Mol Ther Nucleic Acids. (2023) 33:511–28. doi: 10.1016/j.omtn.2023.07.005, 37602275 PMC10432918

[43] Mialon M Camous S Renand G Martal J Ménissier F. Peripheral concentrations of a 60-kDa pregnancy serum protein during gestation and after calving and in relationship to embryonic mortality in cattle. Reprod Nutr Dev. (1993) 33:269–82. doi: 10.1051/rnd:19930309, 8216755

[44] Sasser RG. Detection of pregnancy by radioimmunoassay of a novel pregnancy- specific protein in serum of cows and a profile of serum concentrations during gestation. Biol Reprod. (1986) 35:936–42. doi: 10.1095/biolreprod35.4.936, 3814705

[45] Zoli AP Guilbault LA Delahaut P Ortiz WB Beckers JF. Radioimmunoassay of a bovine pregnancy-associated glycoprotein in serum: its application for pregnancy Diagnosis1. Biol Reprod. (1992) 46:83–92. doi: 10.1095/biolreprod46.1.83, 1547318

[46] Griffin CK Lemley CO Pohler KG Sun X Lear AS. Characterization of placentome vascular perfusion in relation to pregnancy associated glycoproteins throughout gestation in pregnant beef heifers. Theriogenology. (2024) 219:94–102. doi: 10.1016/j.theriogenology.2024.02.020, 38417354

[47] Lobago F Bekana M Gustafsson H Beckers J Yohannes G Aster Y . Serum profiles of pregnancy-associated glycoprotein, Oestrone Sulphate and progesterone during gestation and some factors influencing the profiles in Ethiopian Borana and crossbred cattle. Reprod Domest Anim. (2009) 44:685–92. doi: 10.1111/j.1439-0531.2007.01049.x, 19055565

[48] Doyle RC Herlihy MM Kenneally J Lucy MC Butler ST. An investigation into the factors associated with pregnancy associated glycoproteins in milk in seasonal-calving pasture-based dairy cows. J Dairy Sci. (2025) 108:4332–48. doi: 10.3168/jds.2024-25774, 39892599

[49] Gatea AO Smith MF Pohler KG Egen T Pereira MHC Vasconselos JLM . The ability to predict pregnancy loss in cattle with ELISAs that detect pregnancy associated glycoproteins is antibody dependent. Theriogenology. (2018) 108:269–76. doi: 10.1016/j.theriogenology.2017.12.021, 29275034

[50] Mercadante PM Ribeiro ES Risco C Ealy AD. Associations between pregnancy-associated glycoproteins and pregnancy outcomes, milk yield, parity, and clinical diseases in high-producing dairy cows. J Dairy Sci. (2016) 99:3031–40. doi: 10.3168/jds.2015-10595, 26851856

[51] Pan M Lu J Liu Z Shi H Bai Y Chen P . Integrity of cell-free DNA in maternal plasma extracellular vesicles as a potential biomarker for non-invasive prenatal testing. Int J Gynecol Obstet. (2022) 158:406–17. doi: 10.1002/ijgo.13976, 34626484

[52] Salomon C Scholz-Romero K Sarker S Sweeney E Kobayashi M Correa P . Gestational diabetes mellitus is associated with changes in the concentration and bioactivity of placenta-derived exosomes in maternal circulation across gestation. Diabetes. (2016) 65:598–609. doi: 10.2337/db15-0966, 26718504

[53] Salomon C Torres MJ Kobayashi M Scholz-Romero K Sobrevia L Dobierzewska A . A gestational profile of placental exosomes in maternal plasma and their effects on endothelial cell migration. PLoS One. (2014) 9:e98667. doi: 10.1371/journal.pone.0098667, 24905832 PMC4048215

[54] Drury S Hill M Chitty LS. Cell-free fetal DNA testing for prenatal diagnosis. Adv Clin Chem. (2016, 2016) 76:1–35. doi: 10.1016/bs.acc.2016.05.004 27645814

[55] Zeng Y Qiu Y Jiang W Shen J Yao X He X . Biological features of extracellular vesicles and challenges. Front Cell Dev Biol. (2022) 10:816698. doi: 10.3389/fcell.2022.816698, 35813192 PMC9263222

[56] Perpiñá-Clérigues C Mellado S Galiana-Roselló C Fernández-Regueras M Marcos M García-García F . Novel insight into the lipid network of plasma extracellular vesicles reveal sex-based differences in the lipidomic profile of alcohol use disorder patients. Biol Sex Differ. (2024) 15:10. doi: 10.1186/s13293-024-00584-5, 38273378 PMC10809459

[57] Menon R Debnath C Lai A Guanzon D Bhatnagar S Kshetrapal P . Protein profile changes in circulating placental extracellular vesicles in term and preterm births: a longitudinal study. Endocrinology. (2020) 161:bqaa009. doi: 10.1210/endocr/bqaa009, 31995166 PMC7102872

[58] Salomon C Guanzon D Scholz-Romero K Longo S Correa P Illanes SE . Placental exosomes as early biomarker of preeclampsia: potential role of Exosomal MicroRNAs across gestation. J Clin Endocrinol Metab. (2017) 102:3182–94. doi: 10.1210/jc.2017-00672, 28531338

[59] Pillay P Maharaj N Moodley J Mackraj I. Placental exosomes and pre-eclampsia: maternal circulating levels in normal pregnancies and, early and late onset pre-eclamptic pregnancies. Placenta. (2016) 46:18–25. doi: 10.1016/j.placenta.2016.08.078, 27697217

